# Cost-effectiveness analysis of first-line versus second-line use of CDK4/6 inhibitors combined with endocrine therapy in advanced HR+/HER2- breast cancer in China: based on the SONIA trial

**DOI:** 10.3389/fphar.2025.1700291

**Published:** 2025-11-26

**Authors:** Xiaohu Jin, Zhifeng Li

**Affiliations:** Department of Thyroid and Breast Surgery, Affiliated Hospital 2 of Nantong University (Nantong First People’s Hospital), Nantong, China

**Keywords:** cost-effectiveness analysis, CDK4/6 inhibitors, endocrine therapy, HR+/HER2- breast cancer, SONIA

## Abstract

**Background:**

The optimal sequencing of CDK4/6 inhibitors combined with endocrine therapy for advanced hormone receptor-positive, HER2-negative (HR+/HER2-) breast cancer remains uncertain, particularly in resource-limited settings such as China. This study evaluated the cost-effectiveness of first-line versus second-line CDK4/6 inhibitor use based on the SONIA trial.

**Methods:**

A partitioned survival model was developed to compare costs and effectiveness of first-line (CDK4/6i-first) versus second-line (CDK4/6i-second) CDK4/6 inhibitor strategies among Chinese women with advanced HR+/HER2- breast cancer. Model inputs were derived from the SONIA trial and Chinese healthcare data. Outcomes included total costs, life years (LYs), quality-adjusted life years (QALYs), and incremental cost-effectiveness ratios (ICERs). Both deterministic and probabilistic sensitivity analyses were performed. Scenario analyses incorporated generic drug pricing.

**Results:**

The base-case analysis showed that the CDK4/6i-first strategy yielded 3.07 QALYs at a lifetime cost of CNY 372420.21, compared to 2.86 QALYs and CNY 366445.93 for the CDK4/6i-second strategy. The ICER for first-line CDK4/6 inhibitor use was CNY 28126.33 per QALY, well below the willingness-to-pay (WTP) threshold of CNY 287,247/QALY. Scenario analysis with generics showed an ICER of CNY 198439.62 per QALY. Sensitivity analyses confirmed the robustness of these results.

**Conclusion:**

This study supports the early use of CDK4/6 inhibitors combined with endocrine therapy as a cost-effective strategy for advanced HR+/HER2- breast cancer in China. Continued real-world monitoring is needed to adapt to changes in drug pricing and clinical practice.

## 1 Background

Hormone receptor-positive, human epidermal growth factor receptor 2-negative (HR+/HER2-) advanced breast cancer is the most prevalent subtype of metastatic breast cancer, accounting for over 70% of cases globally (Siegel et al., 2025; NCI, 2025; Howlader et al., 2014). While endocrine therapy (ET) has long served as the cornerstone of treatment, the inevitable development of resistance ultimately limits long-term survival outcomes (Marra et al., 2023). The advent of cyclin-dependent kinase 4/6 (CDK4/6) inhibitors-palbociclib, ribociclib, and abemaciclib-has substantially improved progression-free survival (PFS) and, in some studies, overall survival (OS) when combined with ET in this population (Cristofanilli et al., 2022; Lu et al., 2022; Johnston et al., 2023; Shao et al., 2024). Consequently, major clinical guidelines now recommend CDK4/6 inhibitors plus ET as the preferred first-line regimen (NCCN, 2025). However, the considerable cost of CDK4/6 inhibitors has raised concerns regarding their cost-effectiveness, particularly in settings with constrained healthcare resources (Zhu et al., 2022). In clinical practice, most patients with advanced cancer experience multiple lines of therapy, yet the optimal sequence for administering effective agents remains unclear due to the paucity of prospective randomized evidence. Whether CDK4/6 inhibitors should be initiated in the first-line setting or reserved for use upon progression after initial ET is a key question-particularly given the significant financial and toxicity burdens associated with prolonged CDK4/6 inhibitor exposure.

The phase Ⅲ SONIA trial (NCT03425838) directly addressed this issue by comparing early (first-line) versus delayed (second-line) use of CDK4/6 inhibitors in patients with HR+/HER2− advanced breast cancer (Sonke et al., 2024). Among 1,050 previously untreated patients, median progression-free survival after two lines of therapy was 31.0 months in the first-line CDK4/6 inhibitor group and 26.8 months in the second-line group (hazard ratio 0.87; 95% CI 0.74–1.03; *p* = 0.10). Median overall survival was similar between groups (45.9 vs. 53.7 months; hazard ratio 0.98; 95% CI 0.80–1.20; *p* = 0.83). Importantly, first-line CDK4/6 inhibitor use resulted in longer treatment duration and a higher incidence of grade ≥3 adverse events, without a significant improvement in survival or quality of life.

To date, there is a lack of pharmacoeconomic evidence evaluating the cost-effectiveness of first-line versus second-line CDK4/6 inhibitor use based on robust clinical data. This study therefore evaluates the cost-effectiveness of CDK4/6 inhibitors combined with endocrine therapy in the first- versus second-line setting for HR+/HER2- advanced breast cancer, using data from the SONIA trial and focusing on the Chinese healthcare system.

## 2 Methods

### 2.1 Study design

This study was conducted in accordance with the Consolidated Health Economic Evaluation Reporting Standards (CHEERS) guideline (Supplementary File S1) (Husereau et al., 2022). The target population consisted of adult women in China with HR+/HER2- advanced breast cancer who had not received prior treatment for advanced disease. We assumed that these patients had baseline characteristics similar to those enrolled in the SONIA trial (see Supplementary Table S1 for baseline details).

Patients were divided into two groups: the CDK4/6i-first group (receiving an aromatase inhibitor plus a CDK4/6 inhibitor as first-line therapy, followed by fulvestrant upon progression), and the CDK4/6i-second group (receiving an aromatase inhibitor alone as first-line therapy, with a CDK4/6 inhibitor added to fulvestrant upon progression). The specific treatment regimens and dosing schedules for both groups are detailed in Supplementary Table S2.

### 2.2 Model structure

**FIGURE 1:**
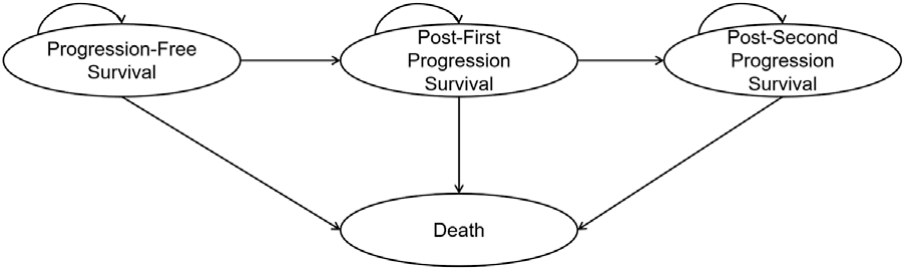
Partitioned survival model structure.

A four-state partitioned survival model was developed to compare the costs and effectiveness of the CDK4/6i-first group and the CDK4/6i-second group. The model consisted of four mutually exclusive health states: progression-free survival, first progression, second progression, and death (Figure 1). The time horizon was set to lifetime, defined as the point at which 99% of the cohort was assumed to have died. The cycle length was set to one treatment cycle (4 weeks). This analysis was conducted from the perspective of the Chinese healthcare system. The primary outcomes included total costs, life years (LYs), quality-adjusted life years (QALYs), and incremental cost-effectiveness ratios (ICER). Both costs and utilities were discounted at an annual rate of 5%. The base-case willingness-to-pay (WTP) threshold was set at three times the *per capita* GDP in China (CNY 287,247/QALY), with an additional threshold of one times *per capita* GDP (CNY 95,749/QALY) also considered (NBS, 2025). Model construction and analysis were performed using R version 4.4.1 and Microsoft Excel. Within R, the “flexsurv” and “survHE” packages were used to reconstruct individual patient data (IPD) and to extrapolate survival outcomes.

### 2.3 Effectiveness

Estimates for PFS, PFS2, and OS were derived from the SONIA trial’s Kaplan-Meier curves. IPD were reconstructed using Guyot’s algorithm with Engauge Digitizer (version 4.1) (Guyot et al., 2012). The reconstructed IPD closely matched the original at-risk population and accurately reproduced the published survival curves. These IPD were used to fit a range of parametric and flexible survival models, including exponential, Weibull, Gompertz, gamma, log-logistic, log-normal, generalized gamma, fractional polynomial, restricted cubic spline, and Royston-Parmar spline models. Model selection was based on the Akaike information criterion (AIC) and visual assessment of fit, with lower AIC values and satisfactory visual concordance indicating a preferred model. Additional details on model selection and external validation are provided in Supplementary File S1 (Supplementary Table S3; Supplementary Figure S1). The four-state partitioned survival model was computed as follows:

${S\_}_{PFS\left(t\right)},{S\_}_{{PFS}_{2}\left(t\right)},{S\_}_{OS\left(t\right)}$ , denote the survivor functions for PFS, PFS2, and OS, respectively.

First, the probability of remaining progression-free at time t is:

$$P\left(Pfs,t\right)=S\_PFS\left(t\right)$$

Accordingly, the probability of being alive after the first progression but before the second progression is:

$$P\left(Post\;1st\;progression,t\right)=S\_{PFS}_{2}\left(t\right)-S\_PFS\left(t\right)$$

In turn, the probability of being alive after the second progression is:

$$P\left(Post\;2nd\;progression,t\right)=S\_OS\left(t\right)-S\_{PFS}_{2}\left(t\right)$$

Finally, the probability of death by time t is:

$$P\left(Death,t\right)=1-S\_OS\left(t\right)$$

To maintain coherence and avoid curve crossing, $S\_OS\left(t\right)\geq S\_{PFS}_{2}\left(t\right)\geq S\_PFS\left(t\right)$ , was enforced through joint or constrained fitting where feasible.

### 2.4 Cost and utility

The economic model incorporated direct medical costs, including drug acquisition, routine follow-up, management of adverse events (AEs), best supportive care, and end-of-life care. Drug unit costs were primarily based on the median 2024 branded drug bidding prices across Chinese provinces, as reported on YAOZH (yaozh.com) (Yaozh.com, 2025). Scenario analyses also considered prices reflecting the availability of generic drugs. For both the intervention and control groups, drug costs were calculated as weighted averages based on the distribution of CDK4/6 inhibitor use among patients in the SONIA Baseline characteristics of patients (Supplementary Table S1). To simplify the analysis, only grade 3 or higher adverse events with an incidence greater than 5% were included; the costs and durations of these events were derived from published sources. All costs were adjusted for inflation to December 2024 using the Consumer Price Index Inflation Calculator (U.S. Bureau of Labor Statistics, 2025). Utility values were obtained from published literature, with disutilities from adverse events applied as negative adjustments. The key model input parameters are summarized in Table 1.

**TABLE 1:** Model input parameters.

Name	Mean	Low	Upper	Distribution	Source
Utility					
Progression-Free Survival (PFS)	0.79	0.71	0.87	beta	Lloyd et al. (2006)
Post-1st progression	0.72	0.65	0.79	beta	
Post-2nd progression	0.45	0.41	0.50	beta	
Cost (CNY)					
Ribociclib succinate tablets (200 mg) [originator]	70.90	63.81	212.06	gamma	Yaozh.com (2025)
Palbociclib Capsules (125 mg) [originator]	203.60	183.24	223.96	gamma	
Palbociclib Capsules (125 mg) [originator and generics]	166.67	165.92	203.60	gamma	
Abemaciclib Tablets (100 mg) [originator]	51.17	46.05	62.32	gamma	
Letrozole Tablets (2.5 mg) [originator]	25.46	22.91	26.50	gamma	
Letrozole Tablets (2.5 mg) [originator and generics]	2.10	1.76	26.49	gamma	
Fulvestrant Injection (250 mg) [originator]	2306.00	2190.70	2533.30	gamma	
Fulvestrant Injection (250 mg) [originator and generics]	427.50	151.30	2306.00	gamma	
Administration (per cycle)	225.70	169.30	282.10	gamma	Lang et al. (2024)
Second-line Treatment Follow-up (per cycle)	1116.50	837.40	1395.60	gamma	
First-line Treatment Follow-up (per cycle)	894.48	670.86	1118.11	gamma	Jia et al. (2025), Lang et al. (2024)
Palliative Care	12732.50	9549.40	15915.60	gamma	Lang et al. (2024)
Best Supportive Care	5427.90	4070.90	6784.90	gamma	
Risk of adverse events in CDK4/6i-first group					
Neutropenia	0.60	0.54	0.66	beta	Sonke et al. (2024)
Leukopenia	0.13	0.12	0.14	beta	
Hypertension	0.08	0.07	0.09	beta	
Elevated GGT (Gamma-glutamyl Transferase)	0.08	0.07	0.09	beta	
Anemia	0.06	0.05	0.07	beta	
Thrombocytopenia	0.06	0.05	0.07	beta	
Risk of adverse events in CDK4/6i-second group					
Neutropenia	0.37	0.33	0.41	beta	Sonke et al. (2024)
Leukopenia	0.09	0.08	0.10	beta	
Hypertension	0.06	0.05	0.07	beta	
Elevated GGT	0.06	0.06	0.07	beta	
Anemia	0.07	0.06	0.08	beta	
Disutility of adverse events					
Neutropenia	0.15	0.14	0.17	beta	Shao et al. (2024)
Leukopenia	0.003	0.002	0.003	beta	
Anemia	0.07	0.07	0.08	beta	
Hypertension	0.04	0.03	0.04	beta	Zhao et al. (2024)
Thrombocytopenia	0.06	0.06	0.07	beta	Yue et al. (2024)
Elevated GGT	0.16	0.11	0.20	beta	Zhu et al. (2022)
Cost for adverse events treatment (CNY)					
Neutropenia	2765.64	2074.19	3457.02	gamma	
Leukopenia	3883.56	2912.71	4854.49	gamma	Zhao et al. (2023)
Anemia	8573.03	7715.73	9430.33	gamma	
Hypertension	9.90	7.91	11.89	gamma	Zhao et al. (2024)
Thrombocytopenia	2680.05	2144.05	3216.12	gamma	
Elevated GGT	2181.21	1871.64	3119.35	gamma	Li et al. (2020)
Other					
Time duration of adverse events (days)	21	14	28	gamma	Sartor (2018)
Discount rate	0.05	0.00	0.08	beta	GE (2020)

### 2.5 Sensitivity analysis

Sensitivity analyses were conducted to test the robustness of the base-case results. Deterministic sensitivity analysis (DSA) was performed by varying all parameters within their 95% confidence intervals or by ±20% of the base-case values. A gamma distribution was assigned to cost inputs, while beta distributions were used for probabilities, proportions, and utilities. Probabilistic sensitivity analysis (PSA) was carried out using 10,000 Monte Carlo simulations to capture parameter uncertainty. Cost-effectiveness acceptability curves (CEACs) were generated to assess the likelihood of each treatment being cost-effective across different WTP thresholds.

### 2.6 Scenario analysis

Given that the inclusion of generic drugs may significantly impact overall costs and potentially alter cost-effectiveness conclusions, scenario analyses were performed incorporating the prices of domestically available generics in China.

## 3 Results

### 3.1 Base case analysis

The base-case analysis demonstrated that the lifetime treatment cost for the CDK4/6i-first group was CNY 372420.21, with a life expectancy of 4.86 years and a total utility of 3.07 QALYs. In comparison, the CDK4/6i-second group incurred a lifetime cost of CNY 366445.93, with a life expectancy of 4.72 years and a total utility of 2.86 QALYs. The ICER was calculated to be CNY 28126.33 per QALY, which is significantly lower than the willingness-to-pay threshold of three times the *per capita* GDP (CNY 287,247 per QALY). These findings indicate that the intervention is more cost-effective than the comparator. A detailed breakdown of costs and effectiveness can be found in Table 2.

**TABLE 2:** Results of base-case analysis.

Type of cost	CDK4/6i-first group	CDK4/6i-second group
Total cost (CNY)	372420.21	366445.93
PFS drug cost	183491.20	82805.44
PFS management costs	41076.02	24993.17
Post-1st Progression drug cost	36351.62	121117.89
Post-1st Progression management costs	7162.53	21120.18
Post-2nd Progression drug cost	88468.75	101190.33
Post-2nd Progression management costs	3678.66	4207.64
End-of-life cost	9176.71	8906.90
Adverse event treatment cost	3014.72	2104.39
Total Life Years (Years)	4.86	4.72
PFS	3.06	1.86
Post-1st Progression	0.44	1.31
Post-2nd Progression	1.36	1.55
Total Effectiveness (QALY)	3.07	2.86
PFS	2.22	1.35
Post-1st Progression	0.29	0.87
Post-2nd Progression	0.56	0.64

### 3.2 Sensitivity analysis

**FIGURE 2:**
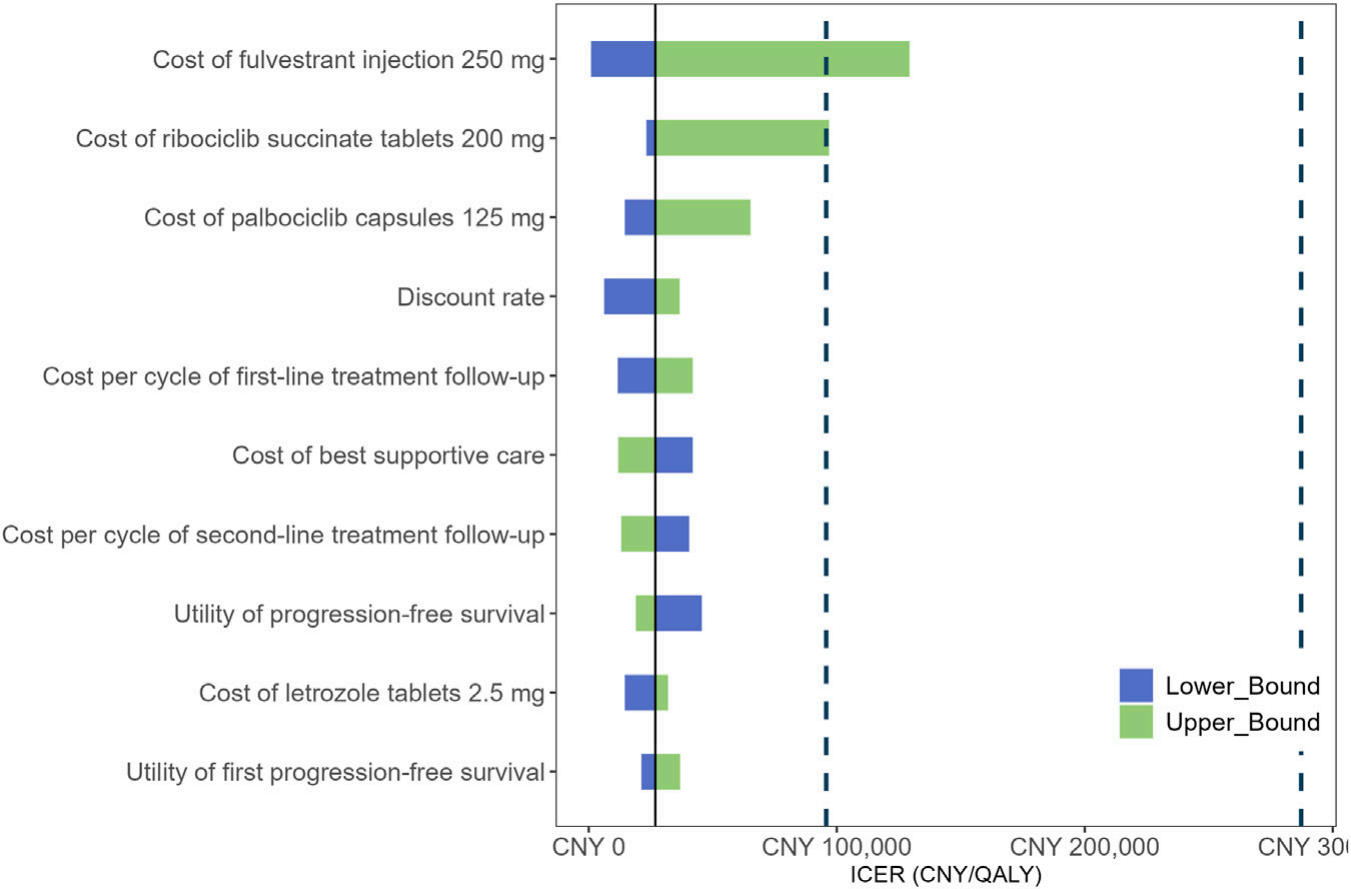
Tornado diagram shows the association of variables with the ICER of CDK4/6i-first group vs. CDK4/6i-second group. ICER, incremental cost-effectiveness ratio; QALY, quality-adjusted life year.

**FIGURE 3:**
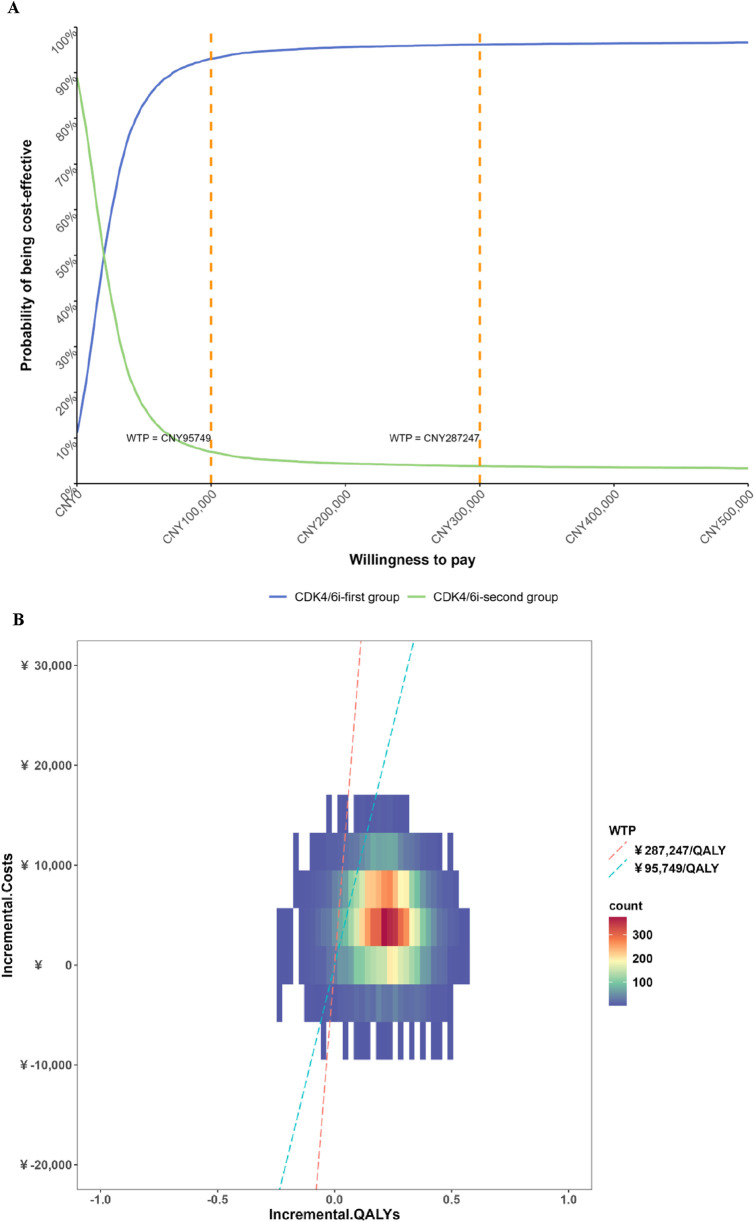
Results of the probabilistic sensitivity analysis cost-effectiveness acceptability curve. **(A)** Cost-effectiveness acceptability curve; **(B)** Probabilistic sensitivity analysis scatter plots.

The results of the DSA are presented in Figure 2. The most influential factors impacting the ICER were identified as the cost of fulvestrant injection 250 mg, the cost of ribociclib succinate tablets 200 mg, and the cost of palbociclib capsules 125 mg. Among these, the cost of fulvestrant injection had the largest effect on the ICER, as fulvestrant represents a substantial component of the treatment regimen and its baseline price is relatively high. Consequently, variations in the price of fulvestrant injection led to the greatest fluctuations in cost-effectiveness outcomes. In addition, the costs of ribociclib and palbociclib also emerged as key drivers of the ICER, reflecting their significant contribution to the overall treatment cost. Other parameters, including the discount rate, the cost per cycle of first- and second-line treatment follow-up, the cost of best supportive care, and the utilities associated with progression-free survival, had comparatively less impact on the ICER. The CEAC is depicted in Figure 3. The PSA showed that at a WTP threshold of CNY 95,749 per QALY, the probability of the intervention being cost-effective was 92.78%. As the WTP threshold increased to CNY 287,247 per QALY, this probability rose sharply, approaching 96.14%.

### 3.3 Scenario analysis

The scenario analysis revealed that the lifetime treatment cost for the CDK4/6i-first group was CNY 295330.97, while that for the CDK4/6i-second group was CNY 253180.66. The life years and effectiveness for both groups remained unchanged compared to the base-case analysis. Under this scenario, the ICER was calculated to be CNY 198439.62 per QALY, which is below the WTP threshold of three times the *per capita* GDP (CNY 287,247 per QALY), but above the threshold of one times the *per capita* GDP. These findings suggest that the intervention remains more cost-effective than the comparator under the scenario assumptions. A detailed breakdown of costs and effectiveness is provided in Table 3.

**TABLE 3:** Results of scenario analysis.

Type of cost	CDK4/6i-first group	CDK4/6i-second group
Total cost (CNY)	295330.97	253180.66
PFS drug cost	133628.32	20786.45
PFS management costs	41076.02	24993.17
Post-1st Progression drug cost	9125.26	69871.60
Post-1st Progression management costs	7162.53	21120.18
Post-2nd Progression drug cost	88468.75	101190.33
Post-2nd Progression management costs	3678.66	4207.64
End-of-life cost	9176.71	8906.90
Adverse event treatment cost	3014.72	2104.39
Total Life Years (Years)	4.86	4.72
PFS	3.06	1.86
Post-1st Progression	0.44	1.31
Post-2nd Progression	1.36	1.55
Total Effectiveness (QALY)	3.07	2.86
PFS	2.22	1.35
Post-1st Progression	0.29	0.87
Post-2nd Progression	0.56	0.64

## 4 Discussion

To our knowledge, this is the first China-based economic evaluation to compare first- versus second-line sequencing of CDK4/6 inhibitors using a whole-disease modeling framework anchored to the SONIA trial. In the context of China’s pricing and reimbursement environment, first-line CDK4/6 inhibition combined with endocrine therapy emerges as a cost-effective strategy and remains so at higher willingness-to-pay thresholds even when generic availability is considered.

This conclusion does not conflict with SONIA’s clinical message that universal first-line use is not mandatory, because economic value is shaped by setting-specific factors. In China, national reimbursement negotiations, volume-based procurement, and provincial tendering have substantially lowered unit prices for CDK4/6 inhibitors and endocrine agents, attenuating the cost penalty associated with longer treatment duration. Earlier initiation also secures access during the period of best performance status and mitigates real-world attrition due to geographic and financial barriers, thereby shifting a greater proportion of survival time into the higher-utility, progression-free state. Standard discounting further increases the present value of these front-loaded health gains. In addition, toxicity management costs constitute a relatively small share of total direct medical expenditures, and first-line follow-up and management typically incur lower per-cycle costs than in later lines, partially offsetting drug spending. Finally, access to, and reimbursement for, certain downstream targeted options remain in evolution, which can limit marginal gains from later-line sequencing and strengthens the case for delivering the most evidence-based combination endocrine therapy earlier in the treatment course.

Unlike most previous cost-effectiveness studies that focused on a single line of therapy, our research incorporated the entire patient treatment pathway (Zhu et al., 2022; Jia et al., 2025; Huang et al., 2021; Jiang et al., 2021; Masurkar et al., 2024; Sun et al., 2025; Pilehvari et al., 2025). Previous analyses typically suggested that first-line CDK4/6 inhibitors plus aromatase inhibitors are more cost-effective than endocrine monotherapy, and that fulvestrant monotherapy is preferable to CDK4/6 inhibitor combinations in the second-line setting. However, single-line evaluations may not reflect the complexity of multi-line treatment in real-world clinical practice. Notably, recent RWE comparing first- versus second-line CDK4/6 inhibitor use indicates a tendency toward improved clinical outcomes with first-line initiation, aligning with the direction of our whole-disease, China-focused economic results (Kimmick et al., 2024; Ravani et al., 2025). Early adoption of high-cost combination regimens may improve short-term PFS, but it can also exhaust healthcare resources upfront, potentially compromising access to subsequent therapies and reducing the efficiency of resource allocation. Our findings emphasize that optimizing cost-effectiveness in a single treatment line does not necessarily translate to optimal value across the whole treatment course. Therefore, pharmacoeconomic evaluations in oncology should be grounded in whole-disease management, considering the interactions between treatment stages and the overall impact on patient outcomes and healthcare resources (Blommestein et al., 2025; Jaber Chehayeb et al., 2022).

Notwithstanding its strengths, this study has limitations. First, survival inputs were reconstructed from published Kaplan–Meier curves and utility weights were sourced from the literature rather than directly from SONIA, and we assumed equal utilities across arms; these choices may introduce bias. Second, because SONIA predominantly involved palbociclib, generalizability to other CDK4/6 inhibitors should be interpreted with caution. Third, the SONIA trial protocol transitioned all patients to second-line therapy upon first-line failure; accordingly, our base-case modeling aligned with this trial-level treatment flow and did not impose additional real-world non-initiation of second-line therapy beyond what is reflected in the survival functions. In routine practice, however, patient attrition between lines (due to clinical deterioration, death, or access barriers) can be higher, especially for the CDK4/6i-second strategy, which could lower true PFS2 drug costs and narrow the cost difference between strategies, potentially increasing the ICER for first-line initiation. Finally, as drug pricing, procurement, and reimbursement continue to evolve-and as access to downstream targeted therapies expands-our conclusions warrant periodic re-evaluation with contemporary real-world data.

## 5 Conclusion

This study provides the first comprehensive economic evaluation of first-line versus second-line use of CDK4/6 inhibitors combined with endocrine therapy for advanced HR+/HER2- breast cancer in China. The findings support the early introduction of CDK4/6 inhibitors as a cost-effective treatment strategy within the Chinese healthcare setting.

## Data Availability

The original contributions presented in the study are included in the article/Supplementary Material, further inquiries can be directed to the corresponding author.

## Abbreviations

AEs, adverse events; AIC, Akaike information criterion; CEACs, Cost-effectiveness acceptability curves; DSA, Deterministic sensitivity analysis; ET, endocrine therapy; HR+/HER2-, hormone receptor-positive, HER2-negative; ICERs, incremental cost-effectiveness ratios; IPD, individual patient data; LYs, life years; OS, overall survival; PFS, progression-free survival; PSA, Probabilistic sensitivity analysis; QALYs, quality-adjusted life years; WTP, willingness-to-pay.
