# Supplementary material for: Cost-effectiveness analysis of first-line versus second-line use of CDK4/6 inhibitors combined with endocrine therapy in advanced HR+/HER2- breast cancer in China: based on the SONIA trial
Source: Front Pharmacol. 2025 Nov 26;16:1700291. doi: 10.3389/fphar.2025.1700291 (PMC12689296; doi:10.3389/fphar.2025.1700291)

**Supplement 2**

[Table S1 Baseline characteristics of patients in SONIA 2](#_Toc205981454)

[Table S2 Comparison of treatment regimens and dosages between intervention and control groups in the SONIA study 3](#_Toc205981455)

[Table S3 The goodness-of-fit results 4](#_Toc205981456)

[Figure S1 Goodness-of-Fit (Visual Validation) of Survival Curves 6](#_Toc205981457)

# Table S1 Baseline characteristics of patients in SONIA

|  | CDK4/6i-first group  (n=524) | CDK4/6i-second group  (n=526) |
| --- | --- | --- |
| **Median age, years (interquartile range)** | 64 (56-71) | 63 (54-71) |
| **ECOG performance status** |  |  |
| 0 | 257 (49%) | 257 (49%) |
| ≥1 | 267 (51%) | 269 (51%) |
| **Menopausal status** |  |  |
| Premenopausal/perimenopausal | 69 (13%) | 76 (14%) |
| Postmenopausal | 455 (87%) | 450 (86%) |
| **Histological subtype** |  |  |
| Lobular | 95(18%) | 86(16%) |
| NST | 394(75%) | 407(77%) |
| **Hormone receptor status** |  |  |
| ER+ | 523 (100%) | 525 (100%) |
| PR+ | 350 (67%) | 353 (67%) |
| **Treatment-free interval** |  |  |
| ≤24 months | 94 (36%) | 91 (36%) |
| >24 months | 163 (64%) | 163 (64%) |
| **Previous (neo)adjuvant therapy** |  |  |
| Chemotherapy | 212 (40%) | 210 (40%) |
| Endocrine therapy | 258 (49%) | 254 (48%) |
| Tamoxifen only | 105 (20%) | 104 (20%) |
| AI only | 32 (6%) | 29 (6%) |
| Sequential | 121 (23%) | 120 (23%) |
| Unknown | 0 (0%) | 1 (0%) |
| **Metastatic site** |  |  |
| Visceral disease | 291 (56%) | 292 (56%) |
| Bone only disease | 91 (17%) | 91 (17%) |
| **Type of CDK4/6i** |  |  |
| Palbociclib | 479 (91%) | 479 (91%) |
| Ribociclib | 42 (8%) | 44 (8%) |
| Abemaciclib | 3 (1%) | 3 (1%) |
| **PIK3CA mutation status†** |  |  |
| Absent | 42(8%) | 68(12%) |
| Present | 33(6%) | 48(9%) |

Note: This table was extracted from SONIA.

† Only patients for whom the mutation status could be retrieved through Palga41 were included. （Casparie, M. et al. Pathology databanking and biobanking in The Netherlands, a central role for PALGA, the nationwide histopathology and cytopathology data network and archive. Cell Oncol. 29, 19–24 (2007).

# Table S2 Comparison of treatment regimens and dosages between intervention and control groups in the SONIA study

| **Treatment regimen** | **CDK4/6i-first group** | **CDK4/6i-second group** |
| --- | --- | --- |
| First-line Treatment | Palbociclib 125 mg orally once daily; 3 weeks on, 1 week off per 28-day cycle +Letrozole 2.5 mg orally once daily (or anastrozole 1 mg)  Ribociclib 600 mg orally once daily; 3 weeks on, 1 week off per 28-day cycle +Letrozole 2.5 mg orally once daily (or anastrozole 1 mg)  Abemaciclib 150mg orally twice daily; per 28-day cycle +Letrozole 2.5 mg orally once daily (or anastrozole 1 mg) | Letrozole 2.5 mg orally once daily (or anastrozole 1 mg) |
| Second-line Treatment | Fulvestrant 500 mg intramuscularly on days 1, 15, and 29, then every 28 days | Fulvestrant 500 mg intramuscularly on days 1, 15, and 29, then every 28 days + CDK4/6 inhibitor (palbociclib or ribociclib) |

# Table S3 The goodness-of-fit results

| Model | LnL | Params | AIC | LnL | Params | AIC | LnL | Params | AIC | LnL | Params | AIC | LnL | Params | AIC | LnL | Params | AIC |
| --- | --- | --- | --- | --- | --- | --- | --- | --- | --- | --- | --- | --- | --- | --- | --- | --- | --- | --- |
|  | CDK4/6i-first group PFS1 | | | CDK4/6i-second group PFS1 | | | CDK4/6i-first group PFS2 | | | CDK4/6i-second group PFS2 | | | CDK4/6i-first group OS | | | CDK4/6i-second group OS | | |
| exp | -149.17 | 1 | 300.34 | -143.68 | 1 | 289.37 | -148.71 | 1 | 299.43 | -148.71 | 1 | 299.43 | -142.69 | 1 | 287.39 | -135.53 | 1 | 273.06 |
| weibull | -147.76 | 2 | 299.51 | -141.75 | 2 | 287.51 | -134.11 | 2 | 272.22 | -134.11 | 2 | 272.22 | -116.26 | 2 | 236.53 | -122.54 | 2 | 249.08 |
| gamma | -146.90 | 2 | 297.80 | -140.73 | 2 | 285.46 | -132.06 | 2 | 268.13 | -132.06 | 2 | 268.13 | -117.42 | 2 | 238.84 | -122.64 | 2 | 249.28 |
| lnorm | -141.14 | 2 | 286.28 | -141.16 | 2 | 286.33 | -131.94 | 2 | 267.88 | -131.94 | 2 | 267.88 | -129.08 | 2 | 262.15 | -129.07 | 2 | 262.14 |
| gompertz | -149.20 | 2 | 302.40 | -143.68 | 2 | 291.35 | -142.62 | 2 | 289.24 | -142.62 | 2 | 289.24 | -120.17 | 2 | 244.33 | -126.31 | 2 | 256.63 |
| llogis | -143.26 | 2 | 290.52 | -139.66 | 2 | 283.33 | -129.95 | 2 | 263.89 | -129.95 | 2 | 263.89 | -117.33 | 2 | 238.65 | -122.49 | 2 | 248.98 |
| gengamma | -141.16 | 3 | 288.32 | -137.50 | 3 | 280.99 | -130.15 | 3 | 266.30 | -130.15 | 3 | 266.30 | -116.25 | 3 | 238.50 | -122.52 | 3 | 251.03 |
| genf | -141.21 | 4 | 290.41 | -137.49 | 4 | 282.98 | -129.90 | 4 | 267.80 | -129.90 | 4 | 267.80 | -115.26 | 4 | 238.52 | -119.36 | 4 | 246.72 |
| FP1-1 | -144.05 | 2 | 292.10 | -137.35 | 2 | 278.70 | -129.01 | 2 | 262.01 | -129.01 | 2 | 262.01 | -120.51 | 2 | 245.03 | -122.55 | 2 | 249.11 |
| FP1-2 | **-140.81** | **2** | **285.61** | -135.65 | 2 | 275.30 | **-128.93** | **2** | **261.87** | **-128.93** | **2** | **261.87** | -131.07 | 2 | 266.15 | -125.25 | 2 | 254.51 |
| FP2-1 | -141.93 | 3 | 289.86 | -135.88 | 3 | 277.75 | -128.39 | 3 | 262.77 | -128.39 | 3 | 262.77 | -113.86 | 3 | 233.73 | -121.10 | 3 | 248.21 |
| FP2-2 | -140.86 | 3 | 287.72 | -135.53 | 3 | 277.07 | -128.49 | 3 | 262.97 | -128.49 | 3 | 262.97 | -113.50 | 3 | 233.00 | -121.10 | 3 | 248.20 |
| RCS1 | -145.86 | 3 | 297.73 | -137.84 | 3 | 281.67 | -131.67 | 3 | 269.35 | -131.67 | 3 | 269.35 | **-112.47** | **3** | **230.93** | **-117.61** | **3** | **241.22** |
| RCS2 | -145.18 | 4 | 298.36 | -137.82 | 4 | 283.65 | -129.92 | 4 | 267.83 | -129.92 | 4 | 267.83 | -112.47 | 4 | 232.93 | -117.00 | 4 | 241.99 |
| RP-hazard-1 | -142.91 | 3 | 291.81 | -131.15 | 7 | 276.29 | -130.68 | 3 | 267.36 | -130.68 | 3 | 267.36 | -116.26 | 2 | 236.53 | -116.54 | 6 | 245.08 |
| RP-hazard-2 | -138.39 | 7 | 290.78 | -130.83 | 6 | 273.67 | -130.08 | 4 | 268.15 | -130.08 | 4 | 268.15 | -115.65 | 3 | 237.29 | -117.56 | 5 | 245.11 |
| RP-odds-1 | -143.26 | 2 | 290.52 | -131.21 | 7 | 276.42 | -129.95 | 2 | 263.89 | -129.95 | 2 | 263.89 | -114.55 | 3 | 235.10 | -116.52 | 6 | 245.04 |
| RP-odds-2 | -137.96 | 7 | 289.92 | **-130.66** | **6** | **273.32** | -129.93 | 3 | 265.87 | -129.93 | 3 | 265.87 | -117.33 | 2 | 238.65 | -117.53 | 5 | 245.07 |
| RP-normal-1 | -141.14 | 2 | 286.28 | -132.25 | 5 | 274.50 | -129.62 | 3 | 265.24 | -129.62 | 3 | 265.24 | -129.08 | 2 | 262.15 | -115.97 | 6 | 243.94 |
| RP-normal-2 | -140.86 | 3 | 287.72 | -132.21 | 6 | 276.41 | -129.45 | 4 | 266.89 | -129.45 | 4 | 266.89 | -128.05 | 5 | 242.06 | -117.05 | 5 | 244.10 |

Note: PFS, progression-free survival; PFS2, Progression-free survival 2; OS, overall survival; LnL, log likelihood; Params, Parameters; AIC, Akaike information criterion; Exp, exponential; lnorm, log normal; llogis, log logistic; gengamma, generalized gamma; FP, fractional polynomial; RCS, restricted cubic spline models; RP, Royston-Parmar models.

Bold data means that this AIC value is one of the smallest top three in this set of data.

# Figure S1 Goodness-of-Fit (Visual Validation) of Survival Curves


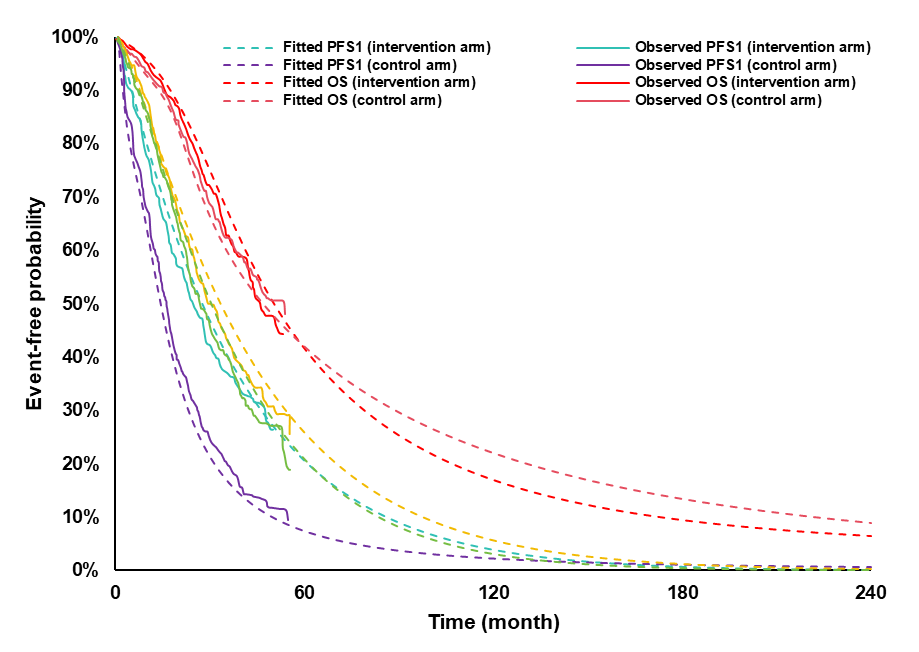

Supplement: Supplementary file 2 [file Supplementaryfile2.docx]
